# Impact of COVID-19 pandemic on gender-based violence in Tunisia

**DOI:** 10.1192/j.eurpsy.2021.2206

**Published:** 2021-08-13

**Authors:** S. Sediri, Y. Zgueb, A. Aissa, U. Ouali, F. Nacef

**Affiliations:** Psychiatry A Department, Razi Hospital, Manouba, Tunisia

**Keywords:** Spouse abuse, domestic violence, lockdown

## Abstract

**Introduction:**

Violence against women is a public health problem worldwide. During humanitarian crises such as wars, violence expands mainly to the detriment of the most vulnerable groups.

**Objectives:**

This study aims to assess the effect of the COVID-19-related lockdown on gender-based violence.

**Methods:**

This study was conducted using an online survey, between April 25 and May 6, 2020. Women were asked about sociodemographic information, lockdown conditions, history involving exposure to violence before and during the COVID-19 lockdown and its types.

**Results:**

The number of included participants was 751. The age ranged from 18 to 69 years. Violence against women increased significantly during the lockdown (from 4.4 to 14.8%; p < 0.001). Psychological abuse was the most frequent type of violence (96%). Almost 90% (n = 98) of those who experienced violence during the lockdown did not seek assistance. Women who had experienced abuse before the lockdown were at an increased risk of violence during lockdown (p < 0.001; OR = 19.34 [8.71–43.00]).

**Conclusions:**

Strengthening strategies to protect women during periods of crisis is urgent. However, a change in mentalities would take more time to set up. Violence against women necessitates a fundamental long-term struggle and practical intervention strategies.

**Disclosure:**

No significant relationships.

